# Correlation between CD44+ cancer stem cell expression and histopathological types of nasopharyngeal carcinoma

**DOI:** 10.12688/f1000research.53643.2

**Published:** 2021-11-03

**Authors:** Muhtarum Yusuf, Indriyadevi Indra, Sri Herawati Juniati, Yussy Afriani Dewi

**Affiliations:** 1 Rhinootolaryngology, Head, and Neck Surgery, Airlangga University, Surabaya, East Java, 60132, Indonesia; 2 Dr. Soetomo General Teaching Hospital, Surabaya, East Java, 60286, Indonesia; 3 Department of Ear-Nose-Throat, Head and Neck Surgery, Hasan Sadikin Hospital, Padjadjaran University, Jl. Dipati Ukur No.35, Bandung, Jawa Barat, 40132, Indonesia

**Keywords:** NPC stem cells, CD44 +, nasopharyngeal carcinoma, histopathology, cancer

## Abstract

**Background: **Nasopharyngeal carcinoma (NPC) recurrency rate is still high despite patients receiving complete treatment. The response to treatment may vary depending on the type of histopathology and Epstein-Barr virus, however the mechanism remains unclear. Recent studies have found that there is a relationship between response to treatment and the presence of cancer stem cells (CSCs). CD44+ cancer stem cells may cause cancer cells to be resistant to treatment. Therefore, this cross-sectional study aims to determine the correlation between CD44 + cancer stem cell expression and the histopathological types of NPC.

**Method:** Samples were obtained from NPC biopsies of type I, II, III patients (based on WHO histopathology criteria), who had not received prior treatment. CD44+ expression was examined using immunohistochemistry methods by staining CD44+ monoclonal antibodies. The degree of CD44+ cell membrane expression was based on the immunoreactive score scale or the Remmele index scale.

**Results: **Most histopathological types were WHO type III (21 patients, 50%), followed by type II (18 patients, 42.86%), and type I (3 patients, 7.14%). CD44 + expression on type I showed one patient had moderate positive and two patients had a high-positive expression. In type II, 10 were moderate positive and eight were high-positive. In type III, one patient was low-positive, 11 were moderate positive and nine patients were high-positive. Statistical analysis showed that the CD44+ expression difference between the three histopathology types were not statistically significant.

**Conclusion:** There were no correlations between CD44 + expression and histopathological type of NPC.

## Introduction

Nasopharyngeal carcinoma (NPC) recurrency rate is still high despite patients receiving complete treatment, such as radiotherapy, chemotherapy, or a combination of both. The responses of NPC to therapy varies greatly and might be associated with the type of histopathology. The histological classification of NPC issued by WHO in 1978 includes: (i) type I as *keratinizing squamous carcinoma*, (ii) type II as *non keratinizing squamous carcinoma*, (iii) type III as *undifferentiated carcinoma.* ^1^ Type II and III World Health Organization (WHO) histopathology types respond more effectively to radiotherapy and have a better locoregional control than type I. ^2^ The difference in therapeutic response was associated with the presence of Epstein-Barr virus (EBV) as a causative factor of NPC, but the mechanism remains unclear. ^3^ Recent studies have shown that there is a relationship between therapeutic response and the presence of cancer stem cells (CSCs). The presence of CSCs can cause cancer cells to be resistant to treatment ^4^^,^^5^ Type II and III WHO histopathology of NPC are closely related to EBV based on the etiology factor of NPC. This was supported by immunoglobulin (IgG and IgA) viral capsid antigen titer increase, early antigen, EBV nuclear antigen (EBNA), however increased titer was not found in type I histopathology of NPC. ^6^ A recent study has shown the important role of EBV in NPC stem cells related to the cancer development. ^7^

CD44+ molecule is an identification marker of NPC stem cells. The study on NPC with positive EBV cell line C666-1 showed that CD44+ cancer stem cells have the capacity to self-renew, differentiate, initiate tumor cells in *vivo,* and they are highly resistant to 5-fluorouracil therapy. ^8^ Ebstein-Barr virus can potentially induced CSCs in EBV-positive NPC by altering the Notch and hedgehog signaling pathways through the *latent membrane protein*-1 (LMP1), LMP2A. ^9^^,^^10^

The purpose of this study was to determine the correlation between CD44+ CSCs expression and the histopathological type in the tissue sample of NPC patients who had received treatment at the department of otolaryngology, head, and neck surgery in the general hospital Dr Soetomo Surabaya (RSUD Dr. Soetomo Surabaya). At present this correlation has not been investigated in this teaching hospital. In a clinical setting the results of this study can be used as a predictor biomarker to assess therapeutic response.

## Methods

In this cross-sectional study 43 tumor tissue samples (n = 43) that were embedded in paraffin blocks were obtained from NPC patients at the otolaryngology, head, and neck surgery department of the RSUD Dr Soetomo Surabaya. These samples were collected from July to September 2016.

We collected subject, age, ethnicity, stage, histopathology and immunoreactive score (IRS) data consecutively according to the inclusion and exclusion criteria (see underlying data: https://figshare.com/s/cd689f9f2918d71671c6). ^11^ After the patient received the explanation and agreed to participate in the study, we collected the histopathological examination numbers from the nasopharyngeal biopsy. We also obtained the paraffin blocks of the NPC patients for further examination of immunohistochemical staining with CD44 monoclonal antibody (Cell marque, USA, Inteli PATH HLX, model number: IPS0001INTL) at the Anatomical Pathology Laboratory, Faculty of Medicine, Airlangga University, Surabaya. To avoid bias, we used positive controls with paraffin block breast carcinoma. CD44 expression was assessed by an anatomical pathologist from the Department of Anatomical Pathology, Faculty of Medicine, Airlangga University, Surabaya.

Tissue samples included in this study were obtained from patients that had not received radiotherapy, chemotherapy, or a combination of both treatments, in addition to the biopsy material having to be sufficient for immunohistochemistry (IHC) examination. The examination of the IHC method was done with a 400× magnification microscope by staining CD44+ monoclonal antibodies (Cell marque, USA, Inteli PATH HLX, model number: IPS0001INTL). ^12^  The assessment of CD44+ cell membrane expression levels were based on the IRS scale or the Remmele scale index ( Table 1). ^13^

**Table 1.** CD44 expression based on IRS.

Positive cells percentage	Color reaction intensity
None: 0	
< 10%: 1	No color reaction: 0
11-50%: 2	Low reaction: 1
51-80%: 3	Moderate reaction: 1
>80%: 4	High reaction: 1

The sample size in this study was calculated using the cross sectional research formula ^31^:

Total sample size = [(Za) . 2p . q] : d2 = (1.962 × 0.5 × 0.5) : 0.152 = 42.38 (rounded to 43 sample).

Note: n = total sample size.

Z = Standard deviation (α = 0.05 with Z = 1.96).

p = 0.5.

Of the 43 samples obtained from the anatomical pathology laboratory, 42 samples met the inclusion criteria, and one damaged sample did not meet the requirements during the immunohistochemical staining process. The population of this study had, 3 people (7.14%) aged 20-29 years, 4 people (9.52%) aged 30-39 years, 14 people (33.33%) aged 40-49 years, 15 people (35.72%) aged 50-59 years and 6 people (14.29%) aged 60-69 years. The male: female ration is 1.3:1, with 24 (57.14%) male patients in the population of the study.

The final score (immunoreactive score/IRS) is the result of multiplying the percentage score of positive cells with the intensity score of brownish-red color on immunoreactive cells.

The degree of expression of CD44 + was categorized as follows:

- Negative expression (-), if the final score is 0
- Low expression (+), if the final score is 1 to 3
- Moderate expression (++), if the final score is 4 to 6
- High expression (+++), if the final score is 7 to 12

NPC histopathology, was divided into three types namely squamous cell carcinoma (WHO type I), nonkeratinizing carcinoma (WHO type II), and undifferentiated carcinoma (WHO type III). ^3^

## Data analysis

Spearman’s statistical test was done to determine the correlation between CD44+ expression and NPC type of histopathology. CD44+ expression and histopathological type were assessed as an ordinal scale with the significance level <0.05.

## Ethical considerations

Patient from the ear nose and throat outpatient unit Dr. Soetomo hospital were given explanation about the research objectives, benefits and examinations carried out. If they were willing to take part in the study, patients were asked to sign a letter of informed consent for the study. Ethical approval (522/Panke.KKE/IX/2016) for this study was obtained by the ethics committee of RSUD Dr Soetomo Surabaya.

## Results

The NPC patients’ distribution data were based on the type of histopathology ( Table 2).

**Table 2.** Distribution of histopathological types.

Histopathology types	N = 42	%
WHO type I	3	7.14
WHO type II	18	42.86
WHO type III	21	50

**Table 3.** Distribution of stage classification.

Stage	N = 42	%
I	1	2.38
II	6	14.29
III	9	21.43
IV	26	61.90

Our results show 21 patients (50%) with WHO-III NPC, followed by 18 (42.86%) with WHO-II, and 3 (7.14%) with WHO-I NPC.

Stage IV was found in 26 (61.90%) patients, followed by stage III in nine (21.43%) patients, stage II in six (14.29%) patients, and stage I in one (2.38%) patient.

CD44 + expression based on the histopathological type of NPC can be seen in Table 4. CD44 + on type I tissue exhibited one moderate positive and two high-positive expressions. In type II tissue, 10 samples showed a moderate positive and eight had high-positive expressions. In type III tissue, one patient had low-positive, 11 had moderate positive and nine had high-positive expressions.

**Table 4.** CD44+ expression based on the histopathological type of NPC.

CD44+ expression (IRS scale)	Histopathological types			N = 42 (%)
	WHO type I	WHO type II	WHO type III	
Low-positive (+)	0	0	1 (4,76%)	1 (2,38%)
Mid-positive (++)	1 (33.33%)	10 (55.56%)	11 (52.38%)	22 (52.38%)
High-positive (+++)	2 (66.67%)	8 (44.44%)	9 (42.86%)	19 (45.24%)
Total	3	18	21	

**Figure 1.**
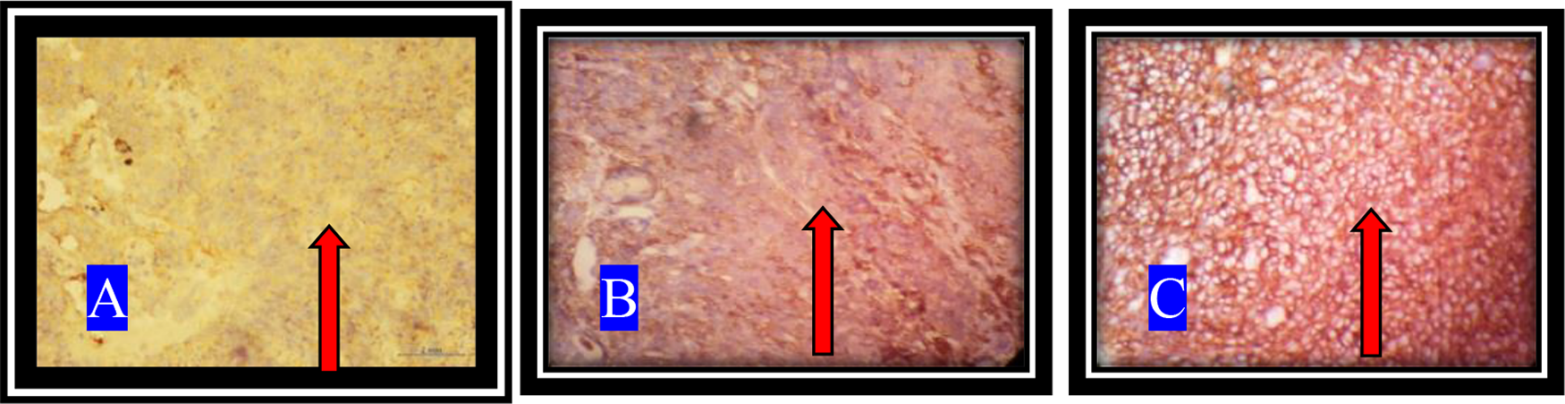
CD44+ expression on NPC tissue. A. The appearance of a brownish-red color with weak intensity which shows a low-positive. B. Medium intensity brownish-red tint indicating a mid-positive. C. A brownish-red high intensity representing a high-positive (arrows).

CD44+ expression with IHC staining with the use of 400x magnification is shown in Figure 1.

Statistical analysis of the correlation between CD44+ expression and the histopathology type of NPC was assessed by Spearman’s correlation test (p = 0.925) with (r) = 0.015. This showed that the high expression of CD44 + was obtained in the histopathology of NPC WHO types I, II, III, however the difference was not statistically significant with p > 0.05.

## Discussion

The purpose of this study was to explain the relationship between CD44+ cancer stem cell expression with the histopathological type of NPC. This relationship can have an important clinical impact since CD44 can be used as a predictor biomarker for assessment of therapeutic response in NPC.

The findings of this study indicate that the majority (15 patients (35.72%)) of NPC were aged 50-59 years. Research from another center at Dr. RSUP. Kariadi Semarang, majority of the NPC patients, 32.80% (128 people), were in the age group 40-49 years, followed by 31.30% that were aged 50-59 years. ^14^As the majority of NPC patients in these studies are above the age of 40, it brings into focus that the theory of carcinogenesis is a multifactorial, multistep process with a delay in diagnosis. ^15^

Patients diagnosed with NPC at a young age tend to experience occupational carcinogenesis. Cancer cells arise from normal cells that undergo transformation to become malignant, due to spontaneous mutation or carcinogen induction. Research has shown that exposure to carcinogens until the emergence of cancer cells can take up to 15-30 years. ^16^

Patients’ sex may be a contributing factor to the incidence of NPC. A total of 24 samples (57.14%) were from male patients in this study, with a ratio of 1.3:1. Research by Roezin (1995) shows that the ratio of NPC sufferers throughout Indonesia was 2.3:1. ^17^ Research in China indicates similar results (2.5:1). ^18^ In Asia, high incidence of NPC in men is thought to be caused by differences in living and work habits, as men are more prone to carcinogen exposure such as smoking, use of firewood, exposure to industrial heat and combustion products, which can cause NPC. ^19^

This research was conducted at Dr. Soetomo Surabaya Hospital, East Java, which is a referral tertiary hospital from Eastern Indonesia, especially the Java region. Therefore, the majority of the research sample are from the Javanese individuals. The research findings also support that the majority of the NPC sufferers (28 samples (66.67%)) were Javanese. Additionally, 61.90% (26 samples) of the samples were NPC stage IV. Research in England also found that stage IV was 52.20%, stage III was 31.30% and stage II was 14.90%, while stage I was not obtained by the study. ^20^ Patients with NPC often seek treatment when this disease is at an advanced stage. Early diagnosis of NPC is challenging as the tumor site is located in a painless area, therefore patients are late to notice this disease. ^21^ Additionally the lack of knowledge about the risks of NPC, along with the distance from the place of treatment, and socioeconomic levels adds to the difficulties of early diagnosis.

Cancer stem cells are a subset of tumor cells that have the ability to self-renew and produce new cells to form tumors. ^22^ The function of cancer stem cells as a progenitor and multipotent cell renewal is for proliferation, differentiation and increasing resistance to cancer therapy. ^23^^,^^24^ NPC has similarities in the type of histopathology and the origin of tumor development between head and neck cancers, including cancer of the oral cavity, pharynx and larynx. In NPC 90% of the histopathological type is squamous cell carcinoma (SCC), which is aggressive and has a high recurrence rate. ^25^ Cluster of differentiation 44 (CD44) is a transmembrane adhesion receptor which is an intermediary for cellular interactions with the tumor microenvirontment that plays a role in the invasion and spread of tumor cells. ^26^^,^^27^ CD44 expression is associated with cellular invasion and metastasis of cancer cells caused by reorganization of the cytoskeleton to facilitate active migration. The expression of CD44 increases the invasion of cancer cells by increasing in proteases and decreasing the expression and enzymatic activity. ^26^

The IHC findings of this study indicate that the increase in CD44 expression is not in parallel with the histopathological types of WHO in NPC, as one WHO type I sample had moderate positive expression (++), and two samples had strong positive expression (+++). In WHO II NPC type 10 samples showed moderate positive expression (++), and eight samples had strong positive expression (+++). In WHO III NPC type one sample had weak positive expression, 11 samples had moderate positive expression (+++), and nine samples had strong positive expression (+++), no negative CD44 expression was found.

Statistical analysis indicated that the correlations between the expression of CD44 cancer stem cells with the histopathological types of NPC is not significant (p = 0.925). This could possibly be due to the sonic hedgehog (Shh) pathway regulation of the CSCs activities, such as self-renewal. ^7^ Positive CD44 expression was found in WHO type I NPC in two patients, WHO II type NPC in ten patients, and WHO III type NPC in nine patients. This suggests that toll-like receptor-3 (TLR3) activates a signaling cascade via the Toll-interleukin-1 receptor [(TIR)] domain-containing-adapter-inducing interferon-β (TRIF), that leads to the activation of the nuclear factor- κB (NF-κB) signaling pathway. ^28^ LMP-1 (latent membrane protein-1) also activates NF-κB and signal transducer and activator of transcription (STAT) signals in EBV-infected epithelial cells via transcription and secretion of interleukin-6 (IL-6). ^29^ Activation of the NF-B signal induces an increase in CD44 expression, which plays a role in the differentiation of NPC cells in WHO I, II and III histopathological types. ^28^^,^^29^

The role of CD44 on cancer development has been found in several studies. Ozman et al. (2014) reported that there was a significant relationship between high CD44 expression and perineural invasion and positive lymph nodes in gastric cancer. Research by Simiuniscu et al., (2012) found a weak staining of CD44 expression in poorly differentiated oral SCC, while moderate and strong expressions were markers of moderately and well differentiated carcinoma. This study found weak positive in one undifferentiated carcinoma sample (WHO type III). In the histopathological type of NPC, 90% is aggressive SCC, with a high recurrence cancer rate. ^25^ In the case of oropharyngeal SCC, it was reported that there was a 39.4% decrease in CD44 isoform expression against the CD44 immunoreactivity criteria, and direct relationship between CD44 expression and cervical lymph node metastases, survival, and recurrence was not found in patients with significantly weak/negative CD44 expression. Those who had weak expression of CD44 had worse free survival compared with positive CD44 expression. ^30^

The limitation of this study was in the sample size. The limitation of this study was in the sample size. The minimum sample was not sufficient because one sample was excluded. CD44 will be a potential marker as follow up measures for NPC patients who have undergone chemoradiation therapy. But from this study it has insignificant result so we cannot recommend CD44 as a targeted therapy and prognostic marker. Furthermore, the researchers only examined the expression of CD44 cancer stem cells without examining CD44 activity. Therefore, the explanation and role of CD44 in NPC stem cells will be more detailed, especially when assessed based on the activity of Shh and Bmi-1 proteins.

## Conclusion

This study has shown that there is no correlation between CD44+ CSCs expression and the histopathological type of NPC. CD44+ expression can not be used as a predictor marker of therapeutic response. Further research is needed, especially with a larger number of samples and multicenter.

## Data availability statement

### Underlying data

Figshare: dataset for The Correlation between CD44+ cancer stem cell expression and histopathological type of nasopharyngeal carcinoma.

DOI: https://figshare.com/s/cd689f9f2918d71671c6. ^11^

The project contains the following underlying data:

CD44_PA_data: Data include subject, age, ethnicity, stage, histopathology and IRS (immunoreactive score *).*

Data are available under the terms of the Creative Commons Zero “No rights reserved” data waiver ( CC0 1.0 Public domain dedication).
