# Supplementary material for: Correlation between CD44+ cancer stem cell expression and histopathological types of nasopharyngeal carcinoma
Source: F1000Res. 2021 Nov 3;10:678. Originally published 2021 Jul 28. [Version 2] doi: 10.12688/f1000research.53643.2 (PMC8581592; doi:10.12688/f1000research.53643.2)
Supplement: Peer review history

# Peer review: Reviewer response for version 2

**DOI:** 10.5256/f1000research.78709.r98927

**Published:** 2021-11-10

**Authors:** Mohd Razif Mohamad Yunus

**Affiliations:** 1 Faculty of Medicine, Department of Otorhinolaryngology, National University of Malaysia, Universiti Kebangsaan Malaysia Medical Centre, Kuala Lumpur, Malaysia

The authors explained well my comments.

Is the work clearly and accurately presented and does it cite the current literature?

Partly

If applicable, is the statistical analysis and its interpretation appropriate?

I cannot comment. A qualified statistician is required.

Are all the source data underlying the results available to ensure full reproducibility?

Partly

Is the study design appropriate and is the work technically sound?

Yes

Are the conclusions drawn adequately supported by the results?

Partly

Are sufficient details of methods and analysis provided to allow replication by others?

Partly

Reviewer Expertise:

Clinical head and neck oncology

I confirm that I have read this submission and believe that I have an appropriate level of expertise to confirm that it is of an acceptable scientific standard.

# Peer review: Reviewer response for version 1

**DOI:** 10.5256/f1000research.57054.r90635

**Published:** 2021-08-02

**Authors:** Mohd Razif Mohamad Yunus

**Affiliations:** 1 Faculty of Medicine, Department of Otorhinolaryngology, National University of Malaysia, Universiti Kebangsaan Malaysia Medical Centre, Kuala Lumpur, Malaysia

It is a good research for the local Indonesian area and data. Reference to their own local data is excellent. The authors have done up to their capabilities at their centre.

 Recommendations:

1. The authors may expand about the limitations of the study and why - possibly due to budget, poor response from the patients etc. as the incidence of NPC in Indonesia is high.
2. May expand the use of having these markers in treatment strategies, possibly as follow up measures.
3. The target sample size should be calculated and included in the methods. This is important to get the significant results of the study. Based on the NPC incidence in Indonesia, the sample size for significant results can be calculated.
4. Multiple centres - collection of sample from other hospital can be considered to get the numbers.
5. It is difficult to give conclusion as stated because the number of sample is not representative of the NPC incidence in Indonesia.

Is the work clearly and accurately presented and does it cite the current literature?

Partly

If applicable, is the statistical analysis and its interpretation appropriate?

I cannot comment. A qualified statistician is required.

Are all the source data underlying the results available to ensure full reproducibility?

Partly

Is the study design appropriate and is the work technically sound?

Yes

Are the conclusions drawn adequately supported by the results?

Partly

Are sufficient details of methods and analysis provided to allow replication by others?

Partly

Reviewer Expertise:

clinical head and neck oncology

I confirm that I have read this submission and believe that I have an appropriate level of expertise to confirm that it is of an acceptable scientific standard, however I have significant reservations, as outlined above.

# Response

**Published:** 2021-08-03

**Authors:** Muhtarum Yusuf

**Affiliations:** Airlangga University, Indonesia

1. The authors may expand about the limitations of the study and why -possibly due to budget, poor response from the patients etc. as the incidence of NPC in Indonesia is high.

 Because the research is limited in time so that the fulfillment of the minimum number of samples to pass the IHC staining on paraffin blocks is quite difficult. In addition, the researchers only examined the expression of CD44 cancer stem cells without examining CD44 activity. Therefore, the explanation and role of CD44 in NPC stem cells will be more detailed, especially when assessed based on the activity of Shh and Bmi-1 proteins.

 2. May expand the use of having these markers in treatment strategies, possibly as follow up measures.

 We might want to use this marker as follow up measures for NPC patients who have undergone chemotherapy. But from this study it has insignificant result so we cannot recommend CD44 as a targeted therapy yet. Therefore, further research is needed, especially those that represent each type of WHO representatively

 3. The target sample size should be calculated and included in the methods. This is important to get the significant results of the study. Based on the NPC incidence in Indonesia, the sample size for significant results can be calculated.

 The sample size in this study was calculated using the cross sectional research formula:

 Total sample size   =  [(Za). ^2^p . q] : d ^2 ^                  

                        = (1,96 ^2^x 0,5 x 0,5) : 0,15 ^2^

**= **42,38 (rounded to 43 sample)

 note:

 n    =  total sample size

 Z    = Standard deviation  (α = 0,05 with Z = 1,96)

 p   =  0,5

 From these calculations, the sample size is sufficient

 4. Multiple centres - collection of sample from other hospital can be considered to get the numbers.

 We are thinking of doing research from multicentre in order to get better external validity

 5. It is difficult to give conclusion as stated because the number of sample is not representative of the NPC incidence in Indonesia.

 This research would be better if it was conducted with a larger number of samples and multicentre. In addition, the sample in this study did not have an equivalent distribution of the number of WHO types. The largest sample in this study was WHO types III-IV with WHO types I and II samples not being adequately represented

 Thanks to reviewer for better advice.

# Peer review: Reviewer response for version 1

**DOI:** 10.5256/f1000research.57054.r90636

**Published:** 2021-08-02

**Authors:** Farhat Farhat

**Affiliations:** 1 Faculty of Medicine, Department of Otorhinolaryngology-Head and Neck Surgery, Universitas Sumatera Utara, Medan, Indonesia

1. The topic for this research is interesting. For now, the recurrency of cancer including NPC is still challenging for clinicians. Exploration about the etiology of the recurrency should be done and the idea to analyze the CD44+ cancer stem cell is interesting. Associating the CD44+ cancer stem cell with the histopathologic type is interesting due to the variation of relation with EBV and sensitivity with radiotherapy. It is good to be focused on population or geographical distribution of the NPC patients. We all know that geographical distribution has high relation with NPC incidence. It can help for mapping the cases.
2. The method of this research is quite clear and complete. The antibody monoclonal that is used for IHC is mentioned. The effort to decrease bias and the references for determining CD44 expression scores are also done and mentioned. Statistical analysis is also clear enough. The ethic approval from ethical committee is also mentioned in the method. 
3. Table and Figure are quite representative. It will be good if the p value is also put on the table. 
4. The insignificant result may be caused by several factors, and I agree that the sample size may also contribute to the result. 

Is the work clearly and accurately presented and does it cite the current literature?

Yes

If applicable, is the statistical analysis and its interpretation appropriate?

Yes

Are all the source data underlying the results available to ensure full reproducibility?

Yes

Is the study design appropriate and is the work technically sound?

Yes

Are the conclusions drawn adequately supported by the results?

Yes

Are sufficient details of methods and analysis provided to allow replication by others?

Yes

Reviewer Expertise:

ENT oncology

I confirm that I have read this submission and believe that I have an appropriate level of expertise to confirm that it is of an acceptable scientific standard.

# Response

**Published:** 2021-08-03

**Authors:** Muhtarum Yusuf

**Affiliations:** Airlangga University, Indonesia

Thank you to the reviewer for the suggestion. Authors think about the possibility of being insignificant for the result of this publication.
